# Long non-coding RNA GAS5 contributes to the progression of nonalcoholic fatty liver disease by targeting the microRNA-29a-3p/NOTCH2 axis

**DOI:** 10.1080/21655979.2022.2026858

**Published:** 2022-03-24

**Authors:** Juanjuan Cui, Yang Wang, Haowei Xue

**Affiliations:** a Department of Stomatology, The First Affiliated Hospital of Anhui Medical University, Hefei, P. R. China; b College of Basic Medical Sciences, Dalian Medical University, Dalian, P. R. China

**Keywords:** NAFLD, progression, lncRNA GAS5, miR-29a-3p, NOTCH2

## Abstract

Long non-coding RNAs (lncRNAs) have been widely recognized as critical players in the development of nonalcoholic fatty liver disease (NAFLD), one of the most prevalent liver diseases globally. In this study, we established a HFD-induced NAFLD mouse model and explored the role of lncRNA GAS5 in NAFLD progression and its possible underlying mechanisms. We showed that NAFLD activity score was elevated in the HFD mice. GAS5 knockdown attenuated HFD-induced hepatic steatosis and lipid accumulation and reduced NAFLD activity score in HFD mice. In addition, GAS5 knockdown reduced serum triglyceride cholesterol levels and inhibited alanine aminotransferase and aspartate aminotransferase activities in HFD mice. Moreover, GAS5 overexpression enhanced NOTCH2 levels in liver cells and promoted NAFLD progression by sponging miR-29a-3p *in vivo*. Furthermore, miR-29a-3p inhibited NAFLD progression by targeting NOTCH2 *in vivo*. Overall, our results indicated that GAS5 acts as a sponge of miR-29a-3p to increase NOTCH2 expression and facilitate NAFLD progression by targeting the miR-29a-3p/NOTCH2 axis and demonstrated a new GAS5-mediated mechanism underlying NAFLD development, suggesting that GAS5 could be a potential therapeutic target of NAFLD.

**Abbreviations:** Alanine aminotransferase: ALT; Aspartate aminotransferase: AST; Enzyme linked immunosorbent assay: ELISA; Hepatocellular carcinoma: HCC; High-fat diet: HFD; Long non-coding RNA: Lnc RNA; Long non-coding RNA GAS5: GAS5; MicroRNAs: MiRNAs; Nonalcoholic fatty liver disease: NAFLD; Quantitative reverse transcription PCRs: RT-qPCRs; siRNA negative control: si-NC; Total cholesterol: TC; Triglyceride: TG

## Background

Nonalcoholic fatty liver disease (NAFLD) is one of the most prevalent hepatic diseases globally and is often correlated with type 2 diabetes, obesity, and harmful diet [1,2]. NAFLD is initiated by irregular triglyceride (TG) increase in the liver and can progress to severe hepatic diseases, including cirrhosis and hepatocellular carcinoma (HCC) [3]. NAFLD accounts for over 13% of hepatocellular carcinoma patients and increases year by year [4]. Therefore, exploring the underlying mechanisms of NAFLD progression and identifying essential treatment targets are crucial to the drug development and therapy of NAFLD [5,6].

Long non-coding RNAs (lncRNAs) modulate gene transcription and translation, as well as chromatin remodeling through binding to RNAs, DNAs, and proteins [7]. Accumulating evidence has indicated lncRNAs as crucial regulators during multiple biological and pathological processes, including cell growth, apoptosis, metastasis, angiogenesis, and liver functions [8,9]. LncRNAs, as regulators of NAFLD progression, are abnormally expressed in NAFLD [10]. It has been reported that lncRNA H19 elevates liver lipogenesis by instantly controlling the miR-130a/PPARγ signaling in NAFLD [11]. The decrease of lncRNA NEAT1 alleviates NAFLD via the mTOR/S6K1 signaling [12]. LncRNA NEAT1/microRNA-140 axis increases NAFLD by disrupting the AMPK/SREBP-1 axis [13]. LncRNA FLRL2 alleviates NAFLD via the Arntl/Sirt1 signaling [14]. LncRNA GAS5 (GAS5) is associated with hepatocellular carcinoma development and is upregulated in the liver tissues of HFD-induced NAFLD mice [15,16]. Nevertheless, whether GAS5 participates in regulating NAFLD pathogenesis has not yet been determined.

MicroRNAs (miRNAs) are another form of extensively studied non-coding RNAs with 20–25 nucleotides. They are well-recognized as important regulators of various biological processes [17]. MiRNAs commonly function through binding to the 3′ untranslated region (3′ UTR) of targeted mRNAs to disrupt their stability or impede their translation [18]. Noteworthy, studies have demonstrated that miRNAs participate in the development of NAFLD. MiR-873-5p modulates the mitochondrial GNMT-Complex II interface, leading to NAFLD [19]. Plasma miR-122 and miR-29a serve as potential markers of NAFLD [20]. Moreover, miR-29a-3p is involved in the modulation of NAFLD. For example, miR-29a-3p controls cholesterol metabolism and triglyceride level by targeting HMGCR in NAFLD [21]. NOTCH2 is a suppressor of transforming growth factor (TGF)-β1 signaling, which is correlated to the maintenance of chronic inflammation and involved in modulating various pathological processes, including NAFLD [22]. Moreover, NOTCH2 has been reported as one of the targets of miR-29a-3p [23,24]. Nevertheless, the connection between GAS5 and miR-29a-3p/NOTCH2 regulatory axis remains unclear. This study aimed to decipher the function of GAS5 during NAFLD pathogenesis and demonstrated the relationship between GAS and the miR-29a-3p/NOTCH2 axis in NAFLD progression.

## Materials and methods

### nonalcoholic fatty liver disease mouse model

The NAFLD mouse model was generated by administrating a high-fat diet (HFD) to C57BL/6 mice. Briefly, male C57BL/6 mice (12–14 weeks old) were assigned into the high-fat diet (HFD) and control groups with 5 mice in each group and maintained at 23 ± 3°C in a humidified atmosphere with a 12-h circadian rhythm and free access to water and high-fat diet (HFD, Dyets Bethlehem, PA, USA) or standard diet (#CE-2, CLEA Japan Inc., Shizuoka, Japan), respectively. The standard diet provided 3.4 kcal per gram and contained 46 g/kg of crude fat, and the HFD provided 5.2 kcal per gram and contained 320 g/kg of lard-based fat. After 56 days, mice in the HF and control groups were weighted 45 ± 4 g and 21 ± 3 g, respectively, and their serum and liver tissues were collected for further analysis. Lentivirus containing shRNAs targeting GAS5 or NOTCH2, miR-29a-3p inhibitors and mimics, and controls were GenePharma (China). For exploring the effects of GAS5 knockdown, NOTCH2 inhibition, and miR-133a-inhibition, lentivirus vector with si-GAS5, si-NOTCH2, and si-NC, miR-133a-inhibitor or its NC, were administrated into mice via tail vein injection. The NAFLD activity score in the mice was quantified as previously reported [25]. The levels of total cholesterol (TC), triglyceride (TG), alanine aminotransferase (ALT), and aspartate Aminotransferase (AST) in serum or liver tissues were analyzed using enzyme-linked immunosorbent assay (ELISA) kits (Applygen, China). The levels of GAS5, NOTCH2, and miR-29a-3p in mouse tissues were detected using qPCR. NOTCH2 protein level was evaluated using Western blotting assay with NOTCH2 antibody (1:1000) from Abcam, USA. Lipid accumulation was analyzed using Oil Red O staining. All animal experiments were approved by the Ethic Committee of the First Affiliated Hospital of Anhui Medical University (No. 34234#HYRE) and operated in compliance with the guidelines of the American Animal Protection Legislation and the Animal Research Reporting In Vivo Experiments.

### Cell culture and transfection

Human normal liver cell line LO2 was obtained from American Type Tissue Culture Collection (ATCC) and cultured in DMEM media (Hyclone, USA) with 10% fetal bovine serum (FBS, Gibco, USA) and 1% streptomycin/penicillin (Sigma, USA) at 37°C with 5% CO_2_. The transfection of vectors carrying GAS5 shRNA, NOTCH2 shRNA, miR-29a-3p inhibitor, and corresponding scramble controls were conducted using Lipofectamine 2000 (Invitrogen, USA) following the manufacturer’s protocol.

### Western blotting assay

Total proteins were obtained from LO2 cells and mouse liver tissues by homogenization in ice-cold RIPA lysis solution (Beyotime, China) and quantified using a BCA kit (Thermo). An equal amount of samples were separated by SDS-PAGE and transferred onto nitrile cellulose membranes. The membranes were blocked in 5% nonfat milk and incubated with first specific primary antibodies against NOTCH2 (1:1000, Santa Cruz, USA) and GAPDH (1:1000, Santa Cruz) at 4°C overnight and then with corresponding secondary anti-mouse or anti-rabbit antibodies (1:2000, Santa Cruz). The protein signals were visualized by an ECL solution in a Gel Imaging system (BD Biosciences, USA) and quantified using ImageJ software.

### Quantitative real-time PCR (qRT-PCR)

Total RNAs were isolated using Trizol reagent (Thermo, USA) following the manufacturer’s description and subjected to cDNA synthesis using a First-strand synthesis kit (Thermo). Relative RNA expression was quantified by qRT-PCR using a SYBR Green Master Mix (Thermo) with U6 and GAPDH as the internal controls for miRNA and mRNA/lncRNA, respectively [26]. The primers used for PCR are 5′-GTGTCTCTCTCTCTCTCTCTCTT-3′ and 5′-CCTCTTCAGCAGTAGCATAGTT-3′ for GAS5, 5′-GTGGCATACTGGGAGGAGAA-3′ and 5′-GATGGAGAAACCAGGGAACA-3′ for NOTCH2, 5′-AAGAAGGTGGTGAAGCAGGC-3′ and 5′-TCCACCACCCAGTTGCTGTA-3′ for GAPDH, 5′-GCACCGTCAAGGCTGAGAAC-3′ and 5′-CAGCCCATCGACTGGTG-3′ for miR-29a-3p, and 5′-AACGCTTCACGAATTTGCGT-3′ and 5′-GCTTCGGCAGCACATATACTAA-3′ for U6.

### Luciferase reporter gene assay

Wild-type and mutated sequences of GAS5 and NOTCH2 were cloned into pGL3-basic vectors purchased from Promega (USA). The obtained vectors were co-transfected with miR-29a-3p mimic or negative control into cells using Lipofectamine 2000. At 48 h of post-transfection, luciferase activity was detected using a dual-luciferase reporter assay kit (Promega) [26].

### RNA pull-down assay

Cells were transfected with biotin-labeled RNAs from GenePharma. At 24 h of post-transfection, cells were lysed and incubated with magnetic beads (Thermo) following the manufacturer’s protocol [27]. The obtained samples were analyzed by qRT-PCR.

### Statistical analysis

Data were shown as mean ± standard deviation (SD) and analyzed by SPSS 22.0 software. The differences were determined by one-way ANOVA or unpaired Student’s *t*-test and were considered significant with *P* < 0.05.

## Results

### LncRNA GAS5 and NOTCH2 are elevated, while miRNA-29a-3p is decreased in the NAFLD mouse model

**Figure 1.**
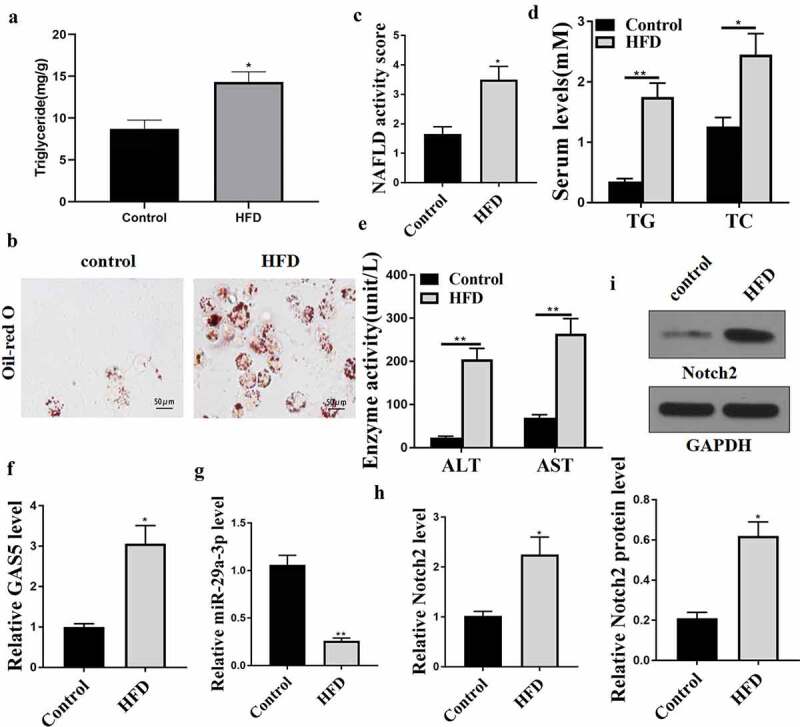
GAS5, miR-29a-3p, and NOTCH2 expression levels are changed in the NAFLD mouse model. (a-h) NAFLD mouse model was established (n = 5). (a) Hepatic TG levels were assessed by ELISA. (b) Oil Red O staining of liver tissues. (c) NAFLD activity score. (d) Serum TC and TG levels were analyzed by ELISA. (e) Serum AST and ALT levels were tested by ELISA. (f) GAS5 expression was determined by qPCR. (g) MiR-29a-3p expression was examined by qPCR. (h) NOTCH2 expression was measured by qPCR. (i) NOTCH2 expression was analyzed by Western blot. Data are presented as mean ± SD. * *P* < 0.05, ** *P* < 0.01.

To evaluate the correlation of GAS5, NOTCH2, and miR-29a-3p with NAFLD, a NAFLD model was established using C57BL/6 mice by feeding HFD. The success of NAFLD mice was evaluated by ELISA and Oil Red staining. The results revealed that the HFD mice had hepatic steatosis (Figure 1(a)), increased lipid accumulation (Figure 1(b)), enhanced NAFLD activity score (Figure 1(c)), and upregulated serum TC (Figure 1(d)), TG (Figure 1(d)), AST (Figure 1(e)), and ALT (Figure 1(e)) levels in comparison with the control mice. RT-qPCR analyses showed that GAS5 RNA level was notably enhanced, whereas miR-29a-3p the level of was declined in liver tissues from the HFD mice (Figure 1(f,g)). Besides, NOTCH2 mRNA and protein levels were enhanced in the liver tissues from the HFD mice (Figure 1(h,i)). Similar changes were also observed in the adipose tissues (Supplement data1). Together, these data imply that GAS5, NOTCH2, and miR-29a-3p may participate in NAFLD modulation.

### LncRNA GAS5 promotes the progression of NAFLD in vivo

**Figure 2.**
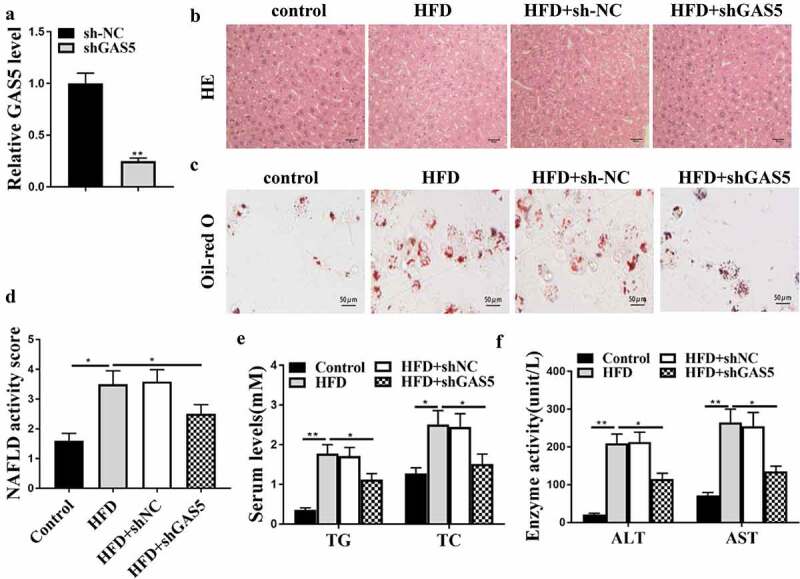
GAS5 promotes NAFLD progression *in vivo*. (a-f) NAFLD mouse model was established. (a) GAS5 expression in mouse liver tissues was determined by qPCR assays. (b) Hepatic steatosis was measured by H&E staining. (c) Oil Red O staining of mouse liver tissues. (d) NAFLD activity score was quantified. (e) Serum TC and TG levels were analyzed by ELISA. (f) Serum AST and ALT levels were tested by ELISA. Data are presented as mean ± SD. * *P* < 0.05, ** *P* < 0.01.

The role of GAS5 in NAFLD development was evaluated in the NAFLD mouse model with GAS5 depletion by injecting lentivirus containing shGAS5 or NC. The effective transfection of sh-GAS5 was determined to evaluate GAS5 RNA level in liver tissues (Figure 2(a)). As shown in (Figure 2(b)), the HFD-induced hepatic steatosis was attenuated by t GAS5 knockdown in the HFD mice. GAS5 depletion reduced lipid accumulation in the mice (Figure 2(c)) and reversed the elevated NAFLD activity score in the HFD mice (*P* < 0.05) (Figure 2(d)). Moreover, GAS5 knockdown attenuated HFD-enhanced serum TG and TC levels (Figure 2(e)) and relieved HFD-caused elevation of serum AST and ALT levels (Figure 2(f)). These results demonstrate that GAS5 promotes NAFLD progression *in vivo*.

### LncRNA GAS5 enhances NOTCH2 expression by sponging miRNA-29a-3p

To evaluate the potential relationships among GAS5, NOTCH2, and miR-29a-3p, we further investigated the correlation among GAS5, miR-29a-3p, and NOTCH2 in LO2 cells using luciferase assay and RT-qPCR.

**Figure 3.**
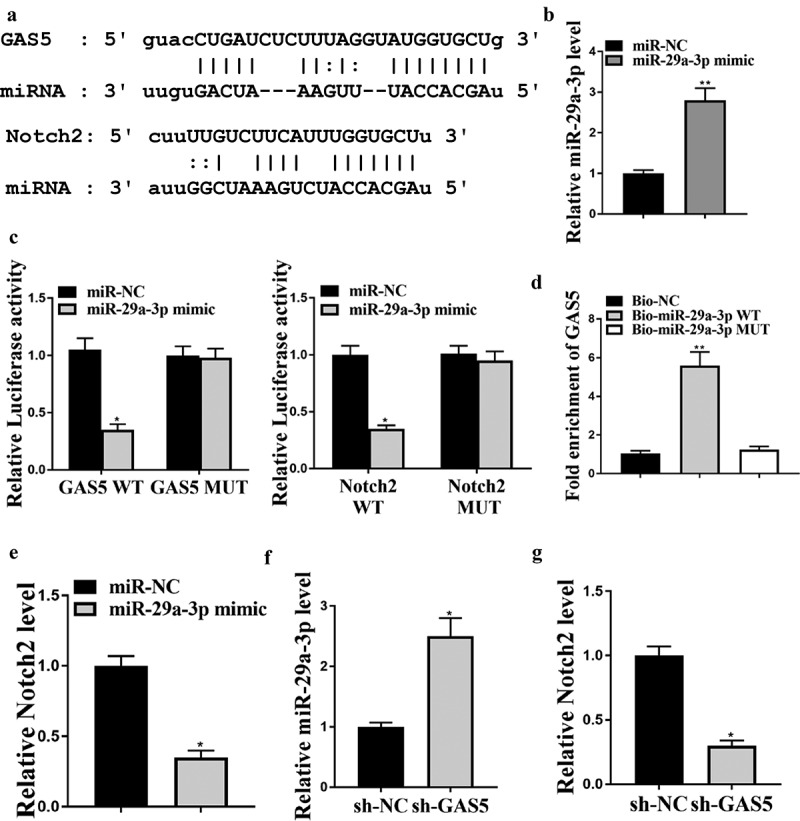
GAS5 enhances NOTCH2 expression by sponging miR-29a-3p. (a) Interactions of miR-29a-3p with GAS5 and NOTCH2 were predicted by bioinformatic analyses using ENCORI (http://starbase.sysu.edu.cn/index.php) and Targetscan (http://www.targetscan.org/vert_72/). (b and c) LO2 cells were treated with control mimic or miR-29a-3p mimic. (b) MiR-29a-3p expression was examined by qPCR. (c) Luciferase activities were determined. (d) Interaction between GAS5 and miR-29a-3p was analyzed by RNA pull-down assays. (e) NOTCH2 expression in liver tissues was measured by qPCR. (f and g) GAS5 shRNA or corresponding control shRNA were transfected in LO2 cells. (f) miR-29a-3p expression was tested by qPCR. (g) NOTCH2 expression was measured by qPCR. Data are presented as mean ± SD. * *P* < 0.05, ** *P* < 0.01.

The potential binding sites of miR-29a-3p on GAS5 and on NOTCH2 3ʹUTR were analyzed by ENCORI and Targetscan online tools, respectively (Figure 3(a)). qRT-PCR analysis indicated that transfection of miR-29a-3p mimic significantly increased miR-29a-3p level in LO2 cells (Figure 3(b)) and miR-29a-3p mimics impaired the luciferase activities of wild-type GAS5 and NOTCH2 vectors but not the vectors containing mutated miR-29a-3p binding sequences (Figure 3(c)). Subsequent RNA pull-down experiment demonstrated that GAS5 interacted with wild-type but not mutant miR-29a-3p (Figure 3(d), *p* < 0.01). Besides, transfection of miR-29a-3p mimics notably downregulated NOTCH2 mRNA level in LO2 cells (Figure 3(e)). GAS5 knockdown significantly elevated miR-29a-3p level (Figure 3(f)) while downregulated NOTCH2 level (Figure 3(g)).

### LncRNA GAS5 promotes NAFLD progression by targeting miR-29a-3p in vivo

**Figure 4.**
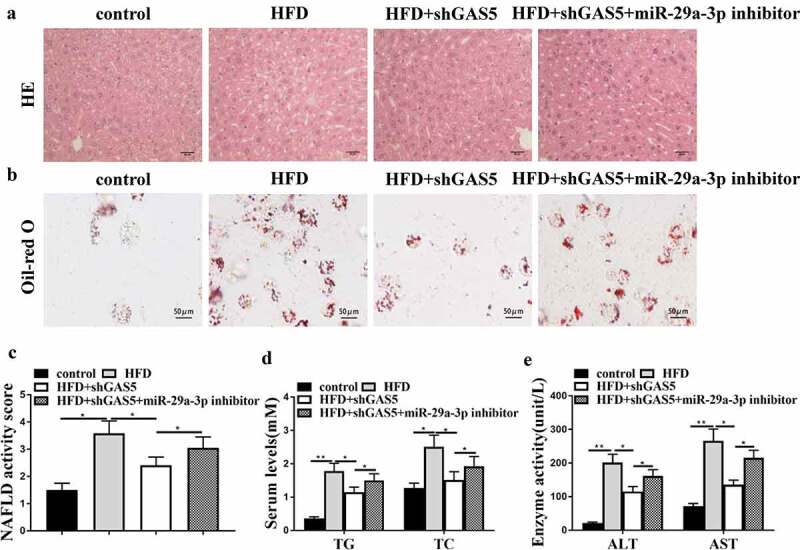
GAS5 promotes NAFLD progression by targeting miR-29a-3p *in vivo*. (a-f) NAFLD mouse model was established. (a) Hepatic steatosis was measured by H&E staining. (b) Oil Red O staining of liver tissues. (c) NAFLD activity score was quantified. (d) Serum TC and TG levels were analyzed by ELISA. (e) Serum AST and ALT levels were tested by ELISA. Data are presented as mean ± SD. * *P* < 0.05, ** *P* < 0.01.

Subsequently, we determined whether GAS5 facilitated NAFLD development by targeting miR-29a-3p in the NAFLD mouse model. For this purpose, lentivirus loaded with sh-GAS5 or negative control with or without miR-29a-3p inhibitor was injected into HFD mice. GAS5 knockdown attenuated HFD-induced hepatic steatosis, and this attenuation was reversed by miR-29a-3p inhibitor (Figure 4(a)). Moreover, GAS5 depletion reduced lipid accumulation of HFD mice, while this reduction was reversed by miR-29a-3p inhibitor (Figure 4(b)). Furthermore, GAS5 depletion reduced NAFLD activity score, while miR-29a-3p inhibitor led to the progression of NAFLD (Figure 4(c)). In addition, GAS5-reduced serum TG, TC, AST, and ALT levels were enhanced by miR-29a-3p inhibitor (Figure 4(d,e)) in HFD mice. These results demonstrate that GAS5 promotes NAFLD progression by targeting miR-29a-3p *in vivo*.

### MiR-29a-3p inhibits NAFLD progression by targeting NOTCH2 in vivo

**Figure 5.**
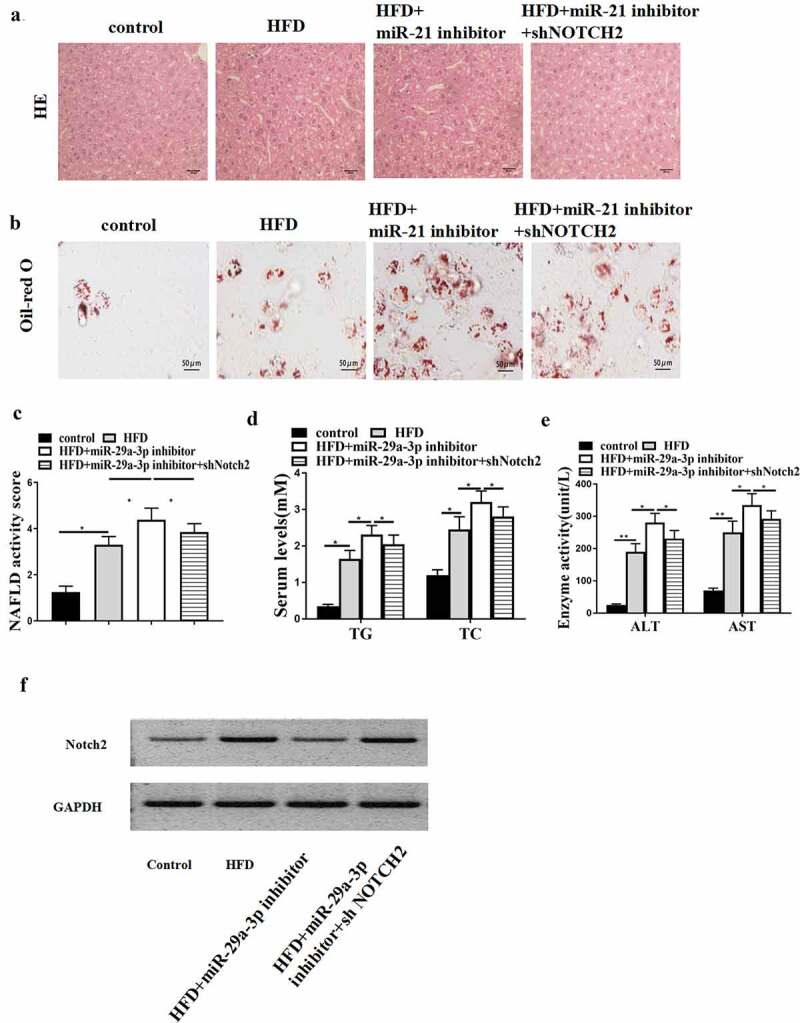
MiR-29a-3p inhibits NAFLD progression by targeting NOTCH2 *in vivo*. (a-f) NAFLD mouse model was established. HFD mice were injected with miR-29a-3p inhibitor or co-injected with miR-29a-3p inhibitor and NOTCH2 shRNA. (a) H&E staining of hepatic steatosis. (b) Oil Red O staining of liver tissues. (c) NAFLD activity score was quantified. (d) Serum TC and TG levels were analyzed by ELISA. (e) Serum AST and ALT levels were tested by ELISA. (f) Notch2 expression was examined using Western blot. Data are presented as mean ± SD. * *P* < 0.05, ** *P* < 0.01.

Next, we explored the possible function of lncRNA GAS5-miR-29a-3p/ NOTCH2 axis in NAFLD progression. MiR-29a-3p inhibitor alone or with NOTCH2 shRNA was injected into HFD mice. HFD-induced hepatic steatosis was enhanced by miR-29a-3p inhibitor, while NOTCH2 depletion reversed this effect (Figure 5(a)). In addition, lipid accumulation in HFD mice was promoted by miR-29a-3p inhibitor but blocked by NOTCH2 knockdown (Figure 5(b)). Moreover, NAFLD activity score was increased by miR-29a-3p inhibitor but reduced by NOTCH2 depletion (*P* < 0.05) (Figure 5(c)). Furthermore, miR-29a-3p inhibitor-enhanced serum TG, TC, AST, and ALT levels were attenuated by NOTCH2 depletion of (Figure 5(d,e)). Furthermore, NOTCH2 protein level was also affected by transfection of shGAS5, miR-21 inhibitor, and shNOTCH2 (Figure 5(f)). These results suggest that miR-29a-3p inhibits NAFLD progression by targeting NOTCH2 *in vivo*.

## Discussion

NAFLD is one of the most frequently occurring chronic hepatic disorders, affecting nearly 20% of the population worldwide [28,29]. It is generally believed that NAFLD consists of four histological steps, including simple steatosis, nonalcoholic steatohepatitis, fibrosis, and cirrhosis. Despite the complicated pathogenesis of NAFLD, lncRNAs have been extensively involved in NAFLD regulation. For instance, lncRNA NEAT1 enhanced liver lipid accumulation by controlling miR-146a-5p/ROCK1 in NAFLD [30]. LncRNA-AK012226 participates in fat accumulation in fatty liver of DB/DB mice and NAFLD cell model [31]. Repression of lncRNA HULC contributes to hepatocyte apoptosis and hepatic fibrosis by repressing MAPK signaling in NAFLD rats [32]. The silencing lncRNA SNHG20 restrains NAFLD progression to hepatocellular carcinoma by modulating liver Kupffer cell polarization [33]. LncRNA Mirt2 upregulates USP10 to repress hepatic steatosis by targeting miR-34a-5p [34]. LncRNA NONMMUT010685 performs a vital function in NAFLD based on microarray analysis [35]. LncRNA NEAT1 controls inflammatory and fibrosis response in NAFLD by mediating miR-506/GLI3 [36]. Moreover, it has been reported that GAS5 inhibits NAFLD development to hepatocellular carcinoma by controlling the Kupffer cell M1/M2 polarization [16]. Our results indicated that GAS5 level was elevated in the HFD mouse model, and this elevation was capable of promoting NAFLD development and revealed a novel role of GAS5 during NAFLD development, thereby providing vital evidence to establish the function of lncRNAs in the pathogenesis of NAFLD.

MiRNAs are well accepted as principle regulators of participants of non-coding RNAs in the various disease progression, including NAFLD. It has been revealed that miR-192-5p modulates lipid accumulation in NAFLD by regulating SCD-1 [37]. MiR-122 increases liver lipogenesis in NAFLD by restraining the LKB1/AMPK signaling via targeting Sirt1 [38]. MiRNA-30b controls insulin sensitivity by modulating SERCA2b in NAFLD [39]. MiRNA-132 causes hyperlipidemia and hepatic steatosis through synergistic multitarget destruction [40]. MiRNA-124a repression inhibits NAFLD by impacting liraglutide intervention and upregulating adipose triglyceride lipase [41]. MiRNA-122 mediates vimentin and hypoxia-inducible factor-1 in hepatocytes and is correlated with fibrosis of diet-produced steatohepatitis [42]. MicroRNA-190b regulates insulin sensitivity and lipid metabolism via targeting ADAMTS9 and IGF-1 in NAFLD [43]. miRNA-375 inhibition modulates inflammatory cytokines and adipokines by targeting AdipoR2 in NAFLD [44]. Furthermore, miR-29a-3p is a potential connection of NAFLD and hepatocellular carcinoma by modulating the HBP1/p53/Srebp1c signaling [45]. LncRNA MEG3 functions as a sponge in controlling hepatic lipogenesis via competitively interacting with miR-29a-3p and LRP6 [46]. MiR-29a-3p regulates WNT/β-catenin signaling in NAFLD pathogenesis [47]and is correlated with fibrosis by serving as a plasma signature of fibrotic disease in a NAFLD rat model [48]. Our data demonstrated that GAS5 enhances NOTCH2 expression by sponging miR-29a-3p and promotes NAFLD progression by targeting miR-29a-3p, suggesting that miR-29a-3p is involved in GAS5-modulated NAFLD and supporting the important role of miR-29a-3p in NAFLD as indicated in previous studies.

NOTCH2 has been indicated in the development of hepatic diseases. For example, it is reported that FBXO31 inflects liver fibrogenesis and hepatic stellate cell activation through increasing NOTCH2 ubiquitination [49]. Hepatic Notch2 deletion in mice contributes to aggravating alcoholic liver injury and mechanical liver damage [50]. NOTCH2 modulates compensatory hepatocyte proliferation in the damaged liver of mice and is positively related to more favorable clinical outcomes of hepatocellular carcinoma [51]. MiR-29a-3p2 increases liver fibrosis and stimulates liver stellate cells by modulating NOTCH2 [52]. The elevated NOTCH2 expression is associated with cholestasis-induced liver fibrogenesis [53]. Moreover, NOTCH2 plays a critical role in TGF-β signaling and is recognized as a miR-29a-3p target [23,24]. Significantly, many investigations have proved that TGF-β signaling is essential for modulating fibrogenesis in NAFLD [54,55]. This study revealed that GAS5 enhances NOTCH2 expression by sponging miR-29a-3p and further inhibits NAFLD progression via targeting NOTCH2, consistent with the previously reported role of NOTCH2 in regulating NAFLD.

To summarize, our work deciphered that GAS5 facilitates NAFLD development via regulating the miR-29a-3p/NOTCH2 regulatory axis. These findings may provide novel evidence for underlying the mechanisms that activate NAFLD progression and present GAS5/miR-29a-3p/NOTCH2 regulatory axis as promising therapeutic targets for NAFLD.

## Conclusions

LncRNA GAS5 and NOTCH2 expression levels are increased, and miR-29a-3p is decreased in the NAFLD mouse model. In addition, lncRNA GAS5 may sponge miR-29a-3p to attenuate the inhibitory role of NOTCH2, thereby promoting NAFLD.

## Supplementary Material

Supplemental Material

## References

[1] Neuschwander-Tetri BA. Non-alcoholic fatty liver disease. BMC Med. 2017;15:45. 28241825 10.1186/s12916-017-0806-8 PMC5330146

[2] Cobbina E, Akhlaghi F. Non-alcoholic fatty liver disease (NAFLD) - pathogenesis, classification, and effect on drug metabolizing enzymes and transporters. Drug Metab Rev. 2017;49:197–211. 28303724 10.1080/03602532.2017.1293683 PMC5576152

[3] Maurice J, Manousou P. Non-alcoholic fatty liver disease. Clin Med (Lond). 2018;18:245–250. 29858436 10.7861/clinmedicine.18-3-245 PMC6334080

[4] Katsiki N, Mikhailidis DP, Mantzoros CS. Non-alcoholic fatty liver disease and dyslipidemia: an update. Metabolism. 2016;65:1109–1123. 27237577 10.1016/j.metabol.2016.05.003

[5] Ter Horst KW, Serlie MJ. Fructose consumption, lipogenesis, and non-alcoholic fatty liver disease. Nutrients. 2017;9. DOI:10.3390/nu9090981 PMC5622741 28878197

[6] Bedossa P. Pathology of non-alcoholic fatty liver disease. Liver Int. 2017;37(Suppl 1):85–89. 28052629 10.1111/liv.13301

[7] Bhan A, Soleimani M, Mandal SS. Long noncoding RNA and cancer: a new paradigm. Cancer Res. 2017;77:3965–3981. 28701486 10.1158/0008-5472.CAN-16-2634 PMC8330958

[8] Herbst RS, Morgensztern D, Boshoff C. The biology and management of non-small cell lung cancer. Nature. 2018;553:446–454. 29364287 10.1038/nature25183

[9] Kopp F, Mendell JT. Functional classification and experimental dissection of long noncoding RNAs. Cell. 2018;172:393–407. 29373828 10.1016/j.cell.2018.01.011 PMC5978744

[10] Sun C, Liu X, Yi Z, et al. Genome-wide analysis of long noncoding RNA expression profiles in patients with non-alcoholic fatty liver disease. IUBMB Life. 2015;67:847–852. 26472541 10.1002/iub.1442

[11] Liu J, Tang T, Wang GD, et al. LncRNA-H19 promotes hepatic lipogenesis by directly regulating miR-130a/PPARgamma axis in non-alcoholic fatty liver disease. Biosci Rep. 2019;39(2):BSR20181722. 31064820 10.1042/BSR20181722 PMC6629946

[12] Wang X. Down-regulation of lncRNA-NEAT1 alleviated the non-alcoholic fatty liver disease via mTOR/S6K1 signaling pathway. J Cell Biochem. 2018;119:1567–1574. 28771824 10.1002/jcb.26317

[13] Sun Y, Song Y, Liu C, et al. LncRNA NEAT1-MicroRNA-140 axis exacerbates nonalcoholic fatty liver through interrupting AMPK/SREBP-1 signaling. Biochem Biophys Res Commun. 2019;516:584–590. 31239155 10.1016/j.bbrc.2019.06.104

[14] Chen Y, Chen X, Gao J, et al. Long noncoding RNA FLRL2 alleviated nonalcoholic fatty liver disease through Arntl-Sirt1 pathway. FASEB J. 2019;33:11411–11419. 31311301 10.1096/fj.201900643RRR

[15] Guo J, Zhou Y, Cheng Y, et al. Metformin-induced changes of the coding transcriptome and non-coding RNAs in the livers of non-alcoholic fatty liver disease mice. Cell Physiol Biochem. 2018;45:1487–1505. 29466788 10.1159/000487575

[16] Wu H, Zhong Z, Wang A, et al. LncRNA FTX represses the progression of non-alcoholic fatty liver disease to hepatocellular carcinoma via regulating the M1/M2 polarization of Kupffer cells. Cancer Cell Int. 2020;20:266. 32595415 10.1186/s12935-020-01354-0 PMC7315496

[17] Lu TX, Rothenberg ME. MicroRNA. J Allergy Clin Immunol. 2018;141:1202–1207. 29074454 10.1016/j.jaci.2017.08.034 PMC5889965

[18] Zhou Q, Huang SX, Zhang F, et al. MicroRNAs: a novel potential biomarker for diagnosis and therapy in patients with non-small cell lung cancer. Cell Prolif. 2017;50:e12394. 10.1111/cpr.12394 PMC6529072 28990243

[19] Fernandez-Tussy P, Fernandez-Ramos D, Lopitz-Otsoa F, et al. miR-873-5p targets mitochondrial GNMT-Complex II interface contributing to non-alcoholic fatty liver disease. Mol Metab. 2019;29:40–54. 31668391 10.1016/j.molmet.2019.08.008 PMC6728756

[20] Jampoka K, Muangpaisarn P, Khongnomnan K, et al. Serum miR-29a and miR-122 as potential biomarkers for non-alcoholic fatty liver disease (NAFLD). Microrna. 2018;7:215–222. 29848284 10.2174/2211536607666180531093302

[21] Sun C, Huang F, Liu X, et al. miR-21 regulates triglyceride and cholesterol metabolism in non-alcoholic fatty liver disease by targeting HMGCR. Int J Mol Med. 2015;35:847–853. 25605429 10.3892/ijmm.2015.2076

[22] Argentou N, Germanidis G, Hytiroglou P, et al. TGF-beta signaling is activated in patients with chronic HBV infection and repressed by SMAD7 overexpression after successful antiviral treatment. Inflamm Res. 2016;65:355–365. 26856334 10.1007/s00011-016-0921-6

[23] Liu G, Friggeri A, Yang Y, et al. miR-21 mediates fibrogenic activation of pulmonary fibroblasts and lung fibrosis. J Exp Med. 2010;207:1589–1597. 20643828 10.1084/jem.20100035 PMC2916139

[24] Chung AC, Dong Y, Yang W, et al. Smad7 suppresses renal fibrosis via altering expression of TGF-beta/Smad3-regulated microRNAs. Mol Ther. 2013;21:388–398. 23207693 10.1038/mt.2012.251 PMC3594008

[25] Santhekadur PK, Kumar DP, Sanyal AJ. Preclinical models of non-alcoholic fatty liver disease. J Hepatol. 2018;68:230–237. 29128391 10.1016/j.jhep.2017.10.031 PMC5775040

[26] Liu S, Xie X, Lei H, et al. Identification of key circRNAs/lncRNAs/miRNAs/mRNAs and pathways in preeclampsia using bioinformatics analysis. Med Sci Monit. 2019;25:1679–1693. 30833538 10.12659/MSM.912801 PMC6413561

[27] Li B, Zhu L, Li L, et al. lncRNA OXCT1-AS1 promotes metastasis in non-small-cell lung cancer by stabilizing LEF1, *In Vitro* and *In Vivo*. Biomed Res Int. 2021;2021:4959381. 34337014 10.1155/2021/4959381 PMC8318766

[28] Chalasani N, Younossi Z, Lavine JE, et al. The diagnosis and management of non-alcoholic fatty liver disease: practice guideline by the American Gastroenterological Association, American Association for the Study of Liver Diseases, and American College of Gastroenterology. Gastroenterology. 2012;142:1592–1609. 22656328 10.1053/j.gastro.2012.04.001

[29] Sattar N, Forrest E, Preiss D. Non-alcoholic fatty liver disease. Bmj. 2014;349:g4596. 25239614 10.1136/bmj.g4596 PMC4168663

[30] Chen X, Tan XR, Li SJ, et al. LncRNA NEAT1 promotes hepatic lipid accumulation via regulating miR-146a-5p/ROCK1 in nonalcoholic fatty liver disease. Life Sci. 2019;235:116829. 31484042 10.1016/j.lfs.2019.116829

[31] Chen X, Xu Y, Zhao D, et al. LncRNA-AK012226 is involved in fat accumulation in db/db mice fatty liver and non-alcoholic fatty liver disease cell model. Front Pharmacol. 2018;9:888. 30135656 10.3389/fphar.2018.00888 PMC6092710

[32] Shen X, Guo H, Xu J, et al. Inhibition of lncRNA HULC improves hepatic fibrosis and hepatocyte apoptosis by inhibiting the MAPK signaling pathway in rats with nonalcoholic fatty liver disease. J Cell Physiol. 2019;234:18169–18179. 30908654 10.1002/jcp.28450

[33] Wang B, Li X, Hu W, et al. Silencing of lncRNA SNHG20 delays the progression of nonalcoholic fatty liver disease to hepatocellular carcinoma via regulating liver Kupffer cells polarization. IUBMB Life. 2019;71:1952–1961. 31408278 10.1002/iub.2137

[34] Zhang B, Li H, Li D, et al. Long noncoding RNA Mirt2 upregulates USP10 expression to suppress hepatic steatosis by sponging miR-34a-5p. Gene. 2019;700:139–148. 30898698 10.1016/j.gene.2019.02.096

[35] Ma TT, Huang C, Ni Y, et al. ATP citrate lyase and LncRNA NONMMUT010685 play crucial role in nonalcoholic fatty liver disease based on analysis of microarray data. Cell Physiol Biochem. 2018;51:871–885. 30466110 10.1159/000495384

[36] Jin SS, Lin XF, Zheng JZ, et al. lncRNA NEAT1 regulates fibrosis and inflammatory response induced by nonalcoholic fatty liver by regulating miR-506/GLI3. Eur Cytokine Netw. 2019;30:98–106. 31957704 10.1684/ecn.2019.0432

[37] Liu XL, Cao HX, Wang BC, et al. miR-192-5p regulates lipid synthesis in non-alcoholic fatty liver disease through SCD-1. World J Gastroenterol. 2017;23:8140–8151. 29290651 10.3748/wjg.v23.i46.8140 PMC5739921

[38] Long JK, Dai W, Zheng YW, et al. miR-122 promotes hepatic lipogenesis via inhibiting the LKB1/AMPK pathway by targeting Sirt1 in non-alcoholic fatty liver disease. Mol Med. 2019;25:26. 31195981 10.1186/s10020-019-0085-2 PMC6567918

[39] Dai LL, Li SD, Ma YC, et al. MicroRNA-30b regulates insulin sensitivity by targeting SERCA2b in non-alcoholic fatty liver disease. Liver Int. 2019;39:1504–1513. 30721562 10.1111/liv.14067

[40] Hanin G, Yayon N, Tzur Y, et al. miRNA-132 induces hepatic steatosis and hyperlipidaemia by synergistic multitarget suppression. Gut. 2018;67:1124–1134. 28381526 10.1136/gutjnl-2016-312869 PMC5969364

[41] Fang QH, Shen QL, Li JJ, et al. Inhibition of microRNA-124a attenuates non-alcoholic fatty liver disease through upregulation of adipose triglyceride lipase and the effect of liraglutide intervention. Hepatol Res. 2019;49:743–757. 30861258 10.1111/hepr.13330

[42] Csak T, Bala S, Lippai D, et al. microRNA-122 regulates hypoxia-inducible factor-1 and vimentin in hepatocytes and correlates with fibrosis in diet-induced steatohepatitis. Liver Int. 2015;35:532–541. 25040043 10.1111/liv.12633 PMC4289469

[43] Xu M, Zheng XM, Jiang F, et al. MicroRNA-190b regulates lipid metabolism and insulin sensitivity by targeting IGF-1 and ADAMTS9 in non-alcoholic fatty liver disease. J Cell Biochem. 2018;119:5864–5874. 29575055 10.1002/jcb.26776

[44] Lei L, Zhou C, Yang X, et al. Down-regulation of microRNA-375 regulates adipokines and inhibits inflammatory cytokines by targeting AdipoR2 in non-alcoholic fatty liver disease. Clin Exp Pharmacol Physiol. 2018;45:819–831. 29569260 10.1111/1440-1681.12940

[45] Wu H, Ng R, Chen X, et al. MicroRNA-21 is a potential link between non-alcoholic fatty liver disease and hepatocellular carcinoma via modulation of the HBP1-p53-Srebp1c pathway. Gut. 2016;65:1850–1860. 26282675 10.1136/gutjnl-2014-308430 PMC4882277

[46] Huang P, Huang FZ, Liu HZ, et al. LncRNA MEG3 functions as a ceRNA in regulating hepatic lipogenesis by competitively binding to miR-21 with LRP6. Metabolism. 2019;94:1–8. 30711569 10.1016/j.metabol.2019.01.018

[47] Wang XM, Wang XY, Huang YM, et al. Role and mechanisms of action of microRNA21 as regards the regulation of the WNT/betacatenin signaling pathway in the pathogenesis of nonalcoholic fatty liver disease. Int J Mol Med. 2019;44:2201–2212. 31638173 10.3892/ijmm.2019.4375 PMC6844630

[48] Takeuchi-Yorimoto A, Yamaura Y, Kanki M, et al. MicroRNA-21 is associated with fibrosis in a rat model of nonalcoholic steatohepatitis and serves as a plasma biomarker for fibrotic liver disease. Toxicol Lett. 2016;258:159–167. 27320964 10.1016/j.toxlet.2016.06.012

[49] He H, Dai J, Feng J, et al. FBXO31 modulates activation of hepatic stellate cells and liver fibrogenesis by promoting ubiquitination of Smad7. J Cell Biochem. 2019. doi:10.1002/jcb.29528. 31680332

[50] Zhu L, Wang L, Wang X, et al. Hepatic deletion of Smad7 in mouse leads to spontaneous liver dysfunction and aggravates alcoholic liver injury. PLoS One. 2011;6:e17415. 21386907 10.1371/journal.pone.0017415 PMC3046253

[51] Feng T, Dzieran J, Gu X, et al. Smad7 regulates compensatory hepatocyte proliferation in damaged mouse liver and positively relates to better clinical outcome in human hepatocellular carcinoma. Clin Sci (Lond). 2015;128:761–774. 25602745 10.1042/CS20140606 PMC10618913

[52] Zhu J, Zhang Z, Zhang Y, et al. MicroRNA-212 activates hepatic stellate cells and promotes liver fibrosis via targeting SMAD7. Biochem Biophys Res Commun. 2018;496:176–183. 29307832 10.1016/j.bbrc.2018.01.019

[53] Seyhan H, Hamzavi J, Wiercinska E, et al. Liver fibrogenesis due to cholestasis is associated with increased Smad7 expression and Smad3 signaling. J Cell Mol Med. 2006;10:922–932. 17125595 10.1111/j.1582-4934.2006.tb00535.x PMC3933087

[54] Ikejima K, Okumura K, Lang T, et al. The role of leptin in progression of non-alcoholic fatty liver disease. Hepatol Res. 2005;33:151–154. 16198623 10.1016/j.hepres.2005.09.024

[55] Yang L, Roh YS, Song J, et al. Transforming growth factor beta signaling in hepatocytes participates in steatohepatitis through regulation of cell death and lipid metabolism in mice. Hepatology. 2014;59:483–495. 23996730 10.1002/hep.26698 PMC3946696

