# Supplementary material for: Long non-coding RNA GAS5 contributes to the progression of nonalcoholic fatty liver disease by targeting the microRNA-29a-3p/NOTCH2 axis
Source: Bioengineered. 2022 Mar 24;13(4):8370–81. doi: 10.1080/21655979.2022.2026858 (PMC9161890; doi:10.1080/21655979.2022.2026858)
Supplement: Supplemental Material [file KBIE_A_2026858_SM4191.zip › supplementary/supp data.pdf]

Supplemental data1

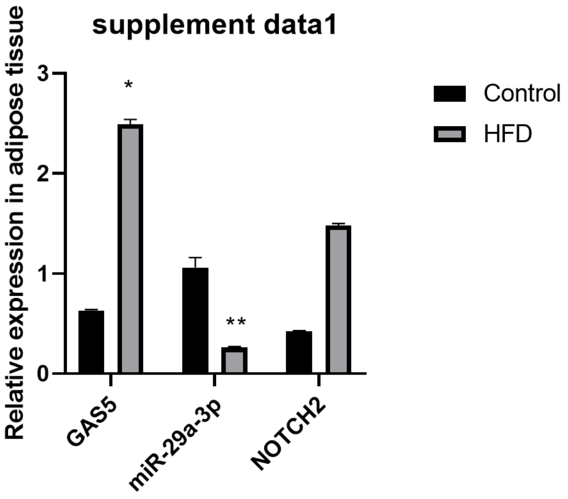

The relative expression of GAS5, miR-29a-3p and NOTCH2 in adipose tissues from control and HFD groups

Expression levels of GAS5, miR-29a-3p, and NOTCH2 in adipose tissues were measured by qPCR. * $P < 0.05$, ** $P < 0.01$. Data are presented as mean ± SD.
